# Lactation-promoting ingredients of *Hemerocallis citrina* Borani and the corresponding mechanisms

**DOI:** 10.3389/fphar.2024.1431856

**Published:** 2024-09-23

**Authors:** Jinpeng Fang, Mengtao Xu, ZhengYue Qiu, Tian Ye, HongLing Jiang, FengYi Qin, Yuan Hu, Faying Jiang, JuHua Zhong, Yishuo Zhu, Lewei Zhao, Xiubin Liu, Jianguo Zeng, Yuqin Xu, Zhixing Qing

**Affiliations:** ^1^ Hunan Key Laboratory of Traditional Chinese Veterinary Medicine, Hunan Agricultural University, Changsha, China; ^2^ Datong Daylily Industial Development Research Institute, Datong, China; ^3^ Department of Pharmacy, The First Hospital of Hunan University of Chinese Medicine, Changsha, China

**Keywords:** *Hemerocallis citrina* Borani, lactation-promoting activity, active ingredient, ultra-performance liquid chromatography-quadrupole-time-of-flight-mass spectrometry, mechanisms

## Abstract

*Hemerocallis citrina* Borani is a traditional folk food used to promote the lactation of *postpartum* mothers in China; however, the active ingredients and corresponding mechanisms are still unknown. In this study, the lactogenic effect of alcoholic and aqueous extracts of *H. citrina* was primarily evaluated, and the aqueous extract (1,000 and 2,000 mg/kg) displayed significant lactation-promoting effects. Three eluates of the aqueous extract (0%, 30%, and 50%HCW) were further evaluated for their lactogenic effect, and 30% and 50% HCW showed significant lactation-promoting activity. Nineteen ingredients, including those with a high content of rutin and isoquercetin, were then identified from 30% and 50%HCW using the ultra-performance liquid chromatography-quadrupole-time-of-flight-mass spectrometry (UPLC-Q-TOF-MS) method. Finally, the lactogenic effect of rutin and isoquercetin was evaluated, and both compounds displayed significant lactation-promoting activity. The mechanisms relative to the lactation-promoting active ingredients for *H. citrina* extracts and compounds are to stimulate the release of prolactin (PRL) and progesterone (P), as well as to induce the expression of prolactin receptor (PRLR) and improve the morphology of mammary tissue. This study first clarified the lactation-promoting active ingredients of *H. citrina* and the corresponding mechanisms, which provide a new insight into the new lactation-promoting drug and promote the high-value utilization of *H. citrina* resources.

## 1 Introduction

Breast milk, which provides infants with the nutrients for growth and development, is unarguably the best source of nutrition for babies (Martin et al., 2016; Savarino et al., 2021). It can also strengthen the immune system, reduce the incidence of disease, and improve the health of babies (Anderson, 2016; Victora et al., 2016). However, the level of breastfeeding is seriously insufficient due to a series of factors, such as the accelerated pace of modern life, increasing work pressure, and an imbalanced diet. The number of people who suffer from breastfeeding shortage is growing, and insufficient breast milk is one of the problems that most mothers have to face in the world (Anderson, 2016). Modern medicine lacks safe and effective therapeutic drugs in the treatment of lactation insufficiency, especially for *postpartum* mothers who are more worried about the side effects of the lactation-promoting drug. Metoclopramide and domperidone are the few effective drugs that can improve lactation in *postpartum* lactation-insufficient mothers (Koch et al., 2019; Tabrizi et al., 2019). However, these drugs have many potential side effects, for example, dizziness, upright hypotension, sleep disorders, palpitations, and other adverse effects. Therefore, there is an urgent need to develop safe and efficient food or drugs to promote lactation for the *postpartum* mother.

*Hemerocallis citrina* Borani (Huang Hua Cai in Chinese) is a plant of the *Hemerocallis* genus in the lily family (Liliaceae). It is also well known as “worry-free grass” or “mother flower” in China. It has been used as a folk food or medicine for a long time (Wang et al., 2017) and has a variety of functions, such as in lactation, antidepressants, insomnia, and laxatives. Its biological activity may be attributed to the flavonols, polyphenols, and fatty acids detected and identified from this plant (Liu et al., 2022). The lactogenic effects of *H. citrina* have also been documented in many ancient books on Chinese medicine, such as “Kunming folk herbs” and “Yunnan herbal medicine.” Modern studies have also shown that fresh *H. citrina* freeze-dried powder and 95% ethanol extract could improve the lactation levels for *postpartum* lactation-deficient rat models (Guo et al., 2023). However, the specific active ingredient and mechanism of lactation-promoting activity of *H. citrina* are still unclear and need further study.

In this study, the lactation activity of aqueous and ethanol extracts of *H. citrina* was evaluated primarily. The result indicated that the aqueous extract displayed significant activity. Column chromatography divided the aqueous extract into three portions, and 30% and 50%HCW showed significant lactation activity. Ultra-performance liquid chromatography-quadrupole-time-of-flight-mass spectrometry (UPLC-Q/TOF-MS) technology was then employed to identify the primary biological activity, and two high-content compounds named rutin and isoquercetin from 30%HCW displayed significant lactation activity. Finally, the mechanism of the lactation-promoting activity of extracts and compounds was clarified by analysis of the level of prolactin (PRL), prolactin receptor (PRLR), and progesterone (P) in *postpartum* lactation-deficient rat models.

## 2 Materials and methods

### 2.1 Chemicals and reagents

Acetonitrile, formic acid, methanol, and xylene were purchased from China National Pharmaceutical Group Corporation (Beijing, China). Bromocriptine mesylate was obtained from Gedeon Richter (Hungary). BuXue ShengRu granules were purchased from Jiuzhitang Co., Ltd. (Changsha, China). The prolactin receptor, prolactin, and progesterone ELISA kit were procured from Shanghai Enzyme-linked Biotechnology Co., Ltd. (Shanghai, China). Furthermore, a 4% paraformaldehyde fixative was purchased from Guangzhou Servicebio Technology Co., Ltd. (Guangzhou, China). EDTA antigen repair solution, citric acid antigen repair solution, PBS, 3% hydrogen peroxide, normal rabbit serum, hematoxylin staining solution, hematoxylin blue return solution, neutral gum, hematoxylin differentiation solution, and histochemistry kit DAB color developer were obtained from Wuhan Saiguo Biotechnology Co., Ltd. (Wuhan, China).

### 2.2 Preparation of *H. citrina* extracts

Sun-dried *H. citrina* flower buds (Mengzihua, 20 Kg) were collected from Qidong County (Hunan Province, China) and were unambiguously identified by Prof. Zhixing Qing (Hunan Agricultural University). Then, 10 kg of *H. citrina* flower buds was divided into two equal parts (each 5 Kg). The extract experiments used water and 70% ethanol as the solvent, respectively. The ratio of material to liquid was 6:1, and the extraction time was 2 h under 100°C. The extraction solvents were concentrated by reducing the pressure and further dried by vacuum. Finally, two different extracts, namely, the aqueous extract (HCW-W) and alcoholic extract (HCE-A), were obtained for the lactation-promoting experiments.

Another 10 kg of *H. citrina* flower buds was extracted using the same method, and a total of 3.72 kg aqueous extract was obtained. The aqueous extract of *H. citrina* was divided into three parts through AB-8 macroporous resin eluated with different concentrations of the ethanol solvent (0%, 30%, and 50%). Finally, three different eluates, namely, 0% ethanol fraction (0%HCE), 30% ethanol fraction (30%HCE), and 50% ethanol fraction (50%HCE), were obtained for further lactation-promoting experiments and UPLC-Q-TOF-MS analysis.

### 2.3 UPLC-Q/TOF-MS conditions

Ultra-high-performance liquid chromatography (UPLC) was used to separate the compounds in *H. citrina*, and quadrupole time-of-flight mass spectrometry (Q-TOF-MS) was used to identify the compounds in *H. citrina* by mass spectrometry (MS) and tandem mass spectrometry (MS/MS). UPLC and Q-TOF-MS conditions are as follows.

Chromatography was performed using an Agilent 1290 UPLC system. An ACQUITY UPLC®BEH-C18 column (100 mm × 2.1 mm, 1.7 µm) was employed as a separation column. The elution solution consisted of 0.1% deionized water (A) and 0.1% acetonitrile (B). The elution program was as follows: 0–20 min, 5–50% B, 20–30 min, and 50–90% B. The column temperature was maintained at 30°C, and the detection wavelength was 254 nm. The rate was set at 0.3 mL/min, and the injection volume was 3 μL.

Mass spectrometric experiments were performed using a 6540 Q-TOF/MS accurate mass spectrometer in a negative mode. The condition of Q-TOF-MS was as follows: drying gas flow rate, 10 L/min; drying gas temperature, 350°C; sheath gas flow rate, 12 L/min; sheath gas temperature, 300°C; atomizing gas pressure, 55 psig; capillary tube voltage, 3,500 V; cone voltage, 100 V; scanning range m/z, 100∼1,700; and secondary fragmentation voltage, 15–30 eV.

### 2.4 Experimental animals

Adult-specific pathogen-free (SPF) grade Sprague–Dawley (SD) rats (9 weeks of age) with a female-to-male ratio of 3:1 and a body weight of 220–235 g were provided by Hunan Slaughter Kingda Laboratory Animal Co., Ltd. [License No. SCXK (Xiang) 2022-0011, Hunan, China]. After mating, all female rats were handled in the same cage for 14 days and then kept separately. After giving birth, 10 infant rats from the litters were selected for the experiment. All animal experiments were conducted in accordance with the guidelines for animal management and experimentation of Hunan Agricultural University. The experiments were approved by the Experimental Animal Ethics Committee of Hunan Agricultural University (HNND-2021-026).

### 2.5 Experimental design and treatments

#### 2.5.1 Evaluation of the lactation-promoting activity of aqueous and alcoholic extracts of *H*. *citrina*

The rats were randomly divided into four groups: the control group (distilled water), model group (bromocriptine), *H. citrina* aqueous extract group (HCW-W), and *H. citrina* alcoholic extract group (HCW-A). Every group contained 3 litters, and every litter included 10 infant rats. The *H. citrina* extract was administered at a dose of 1,000 mg/kg/day by gavage in the HCW-W and HCW-A groups, bromocriptine (10 mg/kg/d) was given to all groups except the control group, and an equal volume of distilled water was given to rats in the control group (Contrera et al., 2004; Dong et al., 2015). All mother rats were administered once in the morning for 12 days.

#### 2.5.2 Optimizing the doses of the *H. citrina* aqueous extract for lactation-promoting activity

The female rats, which have 10 infant rats, were randomly divided into 6 groups: the control group (distilled water), the model group (bromocriptine), the positive group (BuXue ShengRu granule, which is a marketed Chinese medicine used for promoting lactation), and the low-, medium-, and high-dose groups of *H. citrina* (HCE-L, HCE-M, and HCE-H). Every group contained three female rats. The *H. citrina* extract was administered at doses of 500, 1,000, and 2,000 mg/kg/day by gavage daily as low-, medium-, and high-dose groups, respectively. Bromocriptine (4 mg/kg/d) was administered to female rats in all groups except the control group, and rats in the control group were given only an equal volume of distilled water. The positive group was administered 2.6 g/kg by gavage (Chen et al., 2022). All female rats were administered once in the morning for 12 days.

#### 2.5.3 Evaluation of the lactation-promoting activity of 0%HCW, 30%HCW, and 50%HCW

The female rats were randomly divided into six groups: the control group (distilled water), the model group (bromocriptine), the positive group (BuXue ShengRu granules), and the 0%HCW, 30%HCW, and 50%HCW groups. There were 3 female rats in each group, with 10 infant rats for each female rat. Then, 0%HCW, 30%HCW, and 50%HCW were administered corresponding eluates of 1,000 mg/kg/day by gavage, and bromocriptine (4 mg/kg/d) was administered to female rats in the model groups. Female rats in the control group were given only an equal volume of distilled water. The positive group was administered 2.6 mg/kg by gavage. All female rats were administered once in the morning for 12 days.

#### 2.5.4 Evaluation of the lactation-promoting activity of rutin and isoquercetin

The female rats were randomly divided into six groups: the control group (distilled water), the model group (bromocriptine), the positive group (BuXue ShengRu granules), the 30%HCW group, and the rutin and isoquercetin groups. There were 3 female rats in each group, with 10 infant rats for each female rat. The 30%HCW group was administered 1,000 mg/kg/day by gavage, and bromocriptine (4 mg/kg/d) was given to female rats in the model group. The rutin and isoquercetin groups were administered 100 mg/kg by gavage. Female rats in the control group were given only an equal volume of distilled water. The positive group was administered with BuXue ShengRu granules (2.6 g/kg) by gavage. All female rats were administered once in the morning for 12 days.

### 2.6 Measurement indicators

#### 2.6.1 Milk yield

Milk production in female rats is expressed as the change in the weight of all infant rats before and after lactation. Milk production of female rats was calculated using the following formula:

$$\text{Milk production }\left(g/h/d\right)=w3-w2+\left(w1-w2\right)/4,$$

where *W1* is the weight of all infant rats in a group at the time of separation of the mother and pups in the morning; *W2* is the weight of all infant rats after 4 h of separation from the female rat; and *W3* is the weight of all infant rats after 1 h of lactation.

#### 2.6.2 Net weights of the infant rats

The net weight gain of all infant rats for each group was calculated using the following formula:

$$Net\;weight\;gain\;\left(g\right)=W3\;\left(L\right)\;-\;W1\;\left(1\right),$$

where *W3* (L) is the weight of all infant rats on the last day and *W1* (1) is the weight on day 1.

#### 2.6.3 Mammary gland parameters

The mammary gland index in female rats was calculated using the following formula:

$$Mammary\;gland\;index=\frac{W1}{W2}\times 100\%,$$

where *W1* is the weight of mammary tissue (g) and *W2* is the body weight of female rats (g).

#### 2.6.4 Mammary gland structure

Breast tissue was taken and fixed in a 4% neutral paraformaldehyde solution. After 24 h of fixation, tissue paraffin blocks were prepared. After hematoxylin and eosin (H&E) staining and immunohistochemical staining, the structure of the mammary gland was observed using a microscope.

#### 2.6.5 Prolactin, prolactin receptor, and progesterone levels

Blood samples of female rats were collected at room temperature and centrifuged at 3,000 r/min for 15 min at 4°C. The serum was stored at −80°C to quantify the contents of PRL, PRLR, and P using the corresponding ELISA kit, according to the manufacturer’s instructions.

### 2.7 Statistical methods

Data were analyzed by one-way ANOVA using IBM SPSS Statistics 25.0 software. Multiple comparisons were performed using the LSD method for the chi-squared test or the Tamhane method for the missing variance test. *p* < 0.05 was considered statistically significant. The results are expressed as the mean ± standard deviation.

## 3 Results

### 3.1 HCW-W displays significant lactation-promoting activity

**FIGURE 1:**
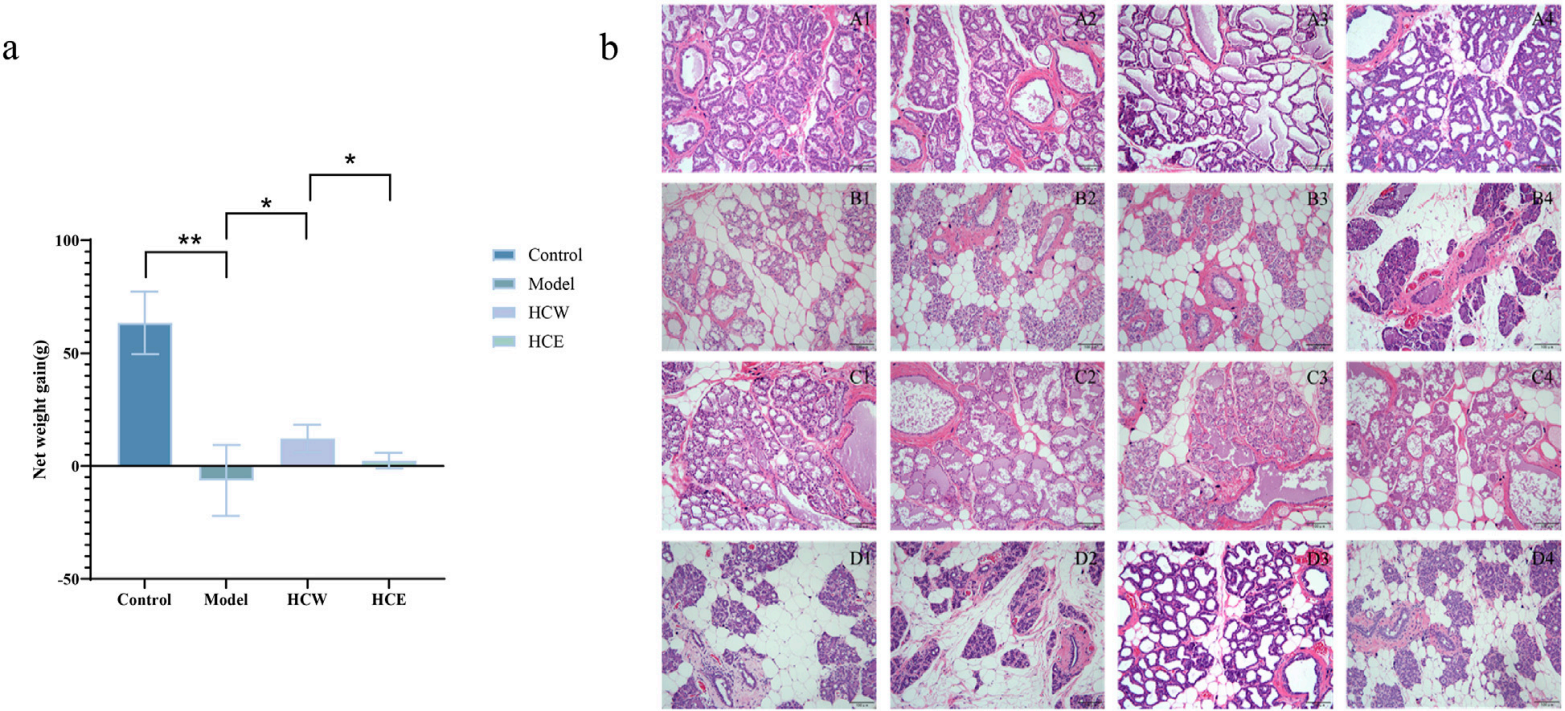
Lactation-promoting activity of aqueous and alcoholic extracts of *H. citrina*. **(a)** Net weight gain of infant rats in different groups; data expressed as the mean ± standard deviation (*p < 0.05 and **p < 0.01; n = 40, from 4 female rats); **(b)** HE staining of mammary gland tissues of female rats from different groups, 20 ×. Among them, **(A)** control group; **(B)** model group (model); **(C)** *H. citrina* aqueous extract group (HCE-W); and **(D)** *H. citrina* alcoholic extract group (HCE-A).

The net weight of infant rats in the model group was significantly decreased compared with the control group (*p* < 0.01) on day 14 after being treated with 4 mg/d/kg bromocriptine (Figure 1A), which indicated that the bromocriptine-induced *postpartum* lactation-deficiency rat model was successful. After treatment with 1,000 mg/d/kg *H. citrina* aqueous extract, the net weight of infant rats was significantly increased compared with the model group (*p* < 0.05). However, the *H. citrina* alcoholic extract did not show lactation-promoting activity. In addition, compared with the *H. citrina* alcoholic extract group, the net weight of infant rats in the *H. citrina* aqueous extract group significantly increased (*p* < 0.05). The above results indicated that the *H. citrina* aqueous extract has significant lactation-promoting activity, which was also in accord with the traditional use and ancient medicinal books (Qing et al., 2021).

Compared with the control group (Figures 1A, B), the bromocriptine group (Figure 1B) showed severe atrophy of the breast tissue, and the normal breast tissue was replaced by a large amount of hyperplastic connective and adipose tissue. The area of the lobules of the breast was reduced, and the lumen of the alveoli in the lobules was significantly smaller. The *H. citrina* aqueous extract group (Figures 1B, C) showed moderate hyperplasia of the mammary lobules and large amounts of secretions in the alveoli and ducts compared with the model group; however, the *H. citrina* alcoholic extract group (Figures 1B–D) showed mild hyperplasia of the glandular follicles and less secretion. The above mammary tissue results indicated that the *H. citrina* aqueous extract has significant lactation-promoting activity. The main chemical constituents of the *H. citrina* extract have been systematically identified in our previous study (Liu et al., 2020).

### 3.2 The medium and high doses of HCW display significant lactation-promoting activity

**FIGURE 2:**
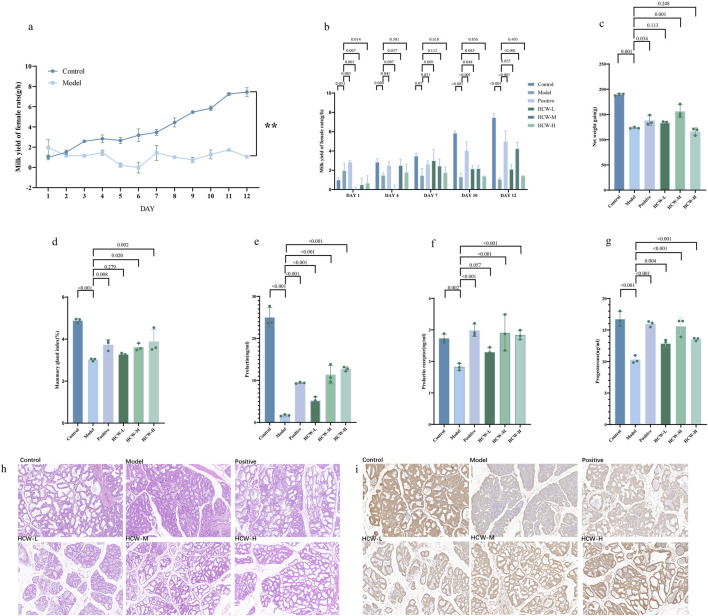
Lactogenic activity of *H. citrina* aqueous extracts at different doses. **(A)** Hourly lactation in the model and control groups. **(B)** Hourly lactation in different groups of female rats for 12 days. **(C)** Net weight gain of each group. **(D)** Mammary index of female rats. Levels of PRL **(E)**, PRLR **(F)**, and P **(G)** in female rats. **(H)** HE staining of mammary tissue in different groups, 20 ×. **(I)** Immunohistochemical staining of PRLR in mammary tissue of different groups.

The lactation-promoting effect of the *H*. *citrina* aqueous extract in different dose groups (500, 1,000, and 2,000 mg/kg) on the bromocriptine-induced *postpartum* lactation-insufficiency rat model was studied. After treatment with 4 mg/kg/day of bromocriptine for 12 days, the milk production in female rats in the model group was significantly decreased compared with the control group (Figure 2A; *p* < 0.01), which indicated that the *postpartum* lactation model was built successfully. On day 12, the milk production in female rats in the positive and medium-dose HCW groups (1,000 mg/kg) was significantly increased compared with the model group (Figure 2B; *p* <0.01), which indicated that the positive drug and medium-dose HCW could reverse the bromocriptine-induced *postpartum* lactation insufficiency. Bromocriptine significantly reduced the body weight gain of the mouse; however, the HCW-M group alleviated the bromocriptine-induced lactation deficiency and increased the body weights of the mouse (Figure 2C). Moreover, the HCW-M group showed the most potent activity for gaining body weight. Bromocriptine significantly reduced the mammary index of the female rats, but the mammary index of the medium- and high-dose groups was significant increased relative to that of the model group (Figure 2D). The bromocriptine significantly decreased the PRL, PRLR, and (P levels. The medium- and high-dose HCW could overcome the effect of bromocriptine, which indicated that the medium- and high-dose HCW could significantly increase the hormone level in female rats and promote lactation (Figures 2E–G).

The model group showed severe atrophy of mammary tissues compared with the control group, and the normal mammary tissue was replaced by a large amount of hyperplastic connective and adipose tissue (Figure 2H). Compared with the model group, the medium- and high-dose HCW group (HCW-M and HCW-H) could increase the mammary lobule area and hyperplasia of intralobular follicles and enlarged the follicular lumens, and most of the follicular lumens returned to normal. The results showed that the HCE-M and HCE-H groups could significantly improve the morphology and structure of the mammary gland tissues of female rats (Figure 2H). The distribution of prolactin receptors in the mammary gland is shown in Figure 2I, where yellow represents the level of prolactin receptors. The level of prolactin receptors in the model group significantly decreased compared with the control group, but after treatment with medium- and high-dose HCW, the content of prolactin receptors was significantly increased. The above results indicated that HCW-M and HCW-H show significant lactation-promoting activity.

### 3.3 30%HCW and 50%HCW display significant lactation-promoting activity

**FIGURE 3:**
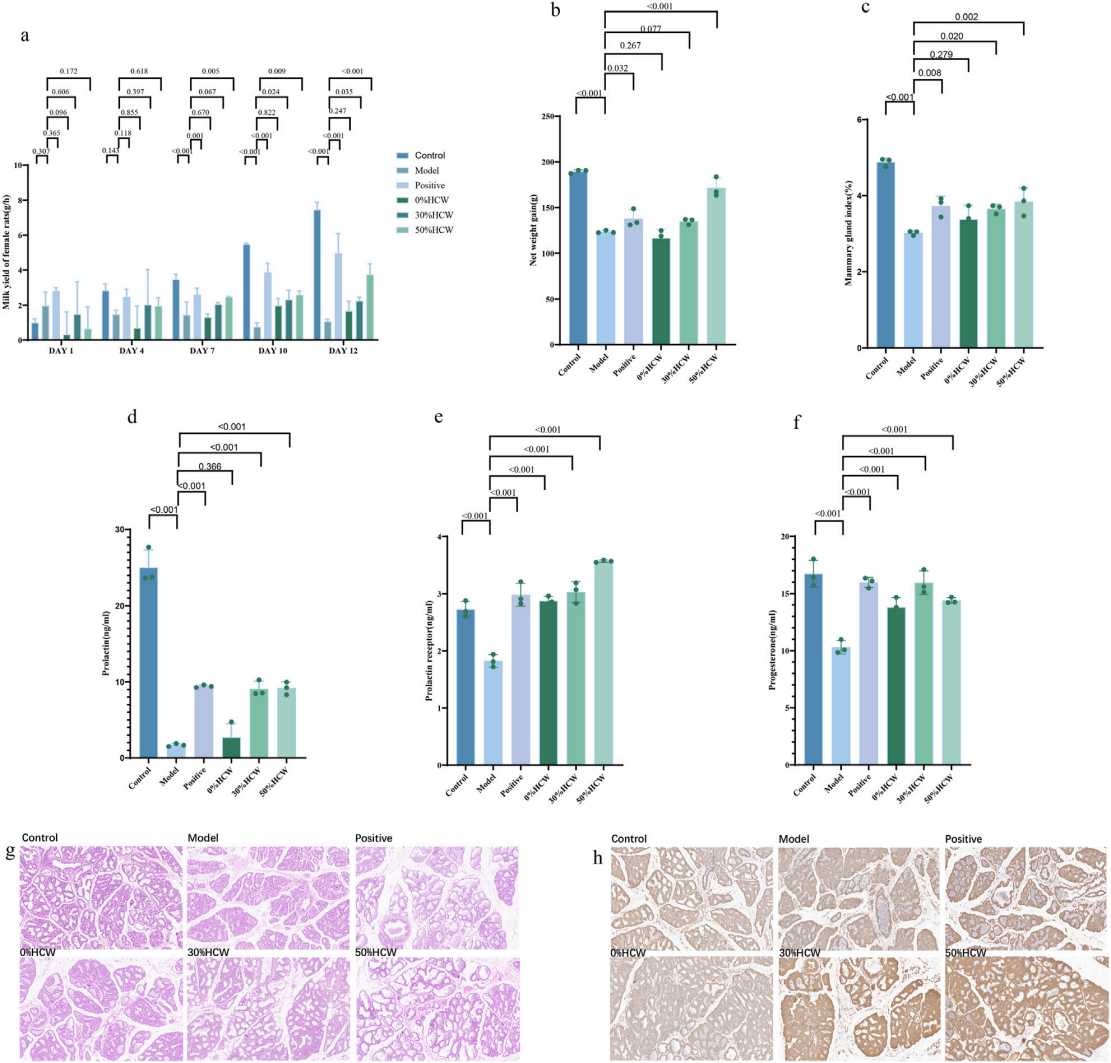
Lactogenic activity of 30%HCW and 50%HCW. **(A)** Hourly lactation in different groups of female rats on days 1, 4, 7, 10, and 12; **(B)** net weight gain of each groups; **(C)** mammary index of female rats; levels of PRL **(D)**, PRLR **(E)**, and P **(F)** in female rats; **(G)** HE staining of mammary tissue of different groups, 20 ×; and **(H)** immunohistochemical staining of PRLR in mammary tissue of different groups.

Three different eluates, namely, 0%HCW, 30%HCW, and 50%HCW, were isolated from the aqueous extract of *H. citrina* using AB-8 macroporous resin, and their lactation-promoting activity was evaluated using the bromocriptine-induced *postpartum* lactation-insufficiency rat model. As shown in Figure 3A, the milk production of female rats in the 30%HCW and 50%HCW groups (1,000 mg/kg) was significantly increased compared with the control group (*p* < 0.05) on days 10 and 12, which indicated that both eluates could reverse the bromocriptine-induced *postpartum* lactation insufficiency. Bromocriptine could significantly reduce the body weight gain of the young rats (*p* < 0.01); however, the 30%HCW and 50%HCW groups alleviated the bromocriptine-induced lactation deficiency of the female rats and increased the body weights of the young rats. Moreover, the 50%HCW group displayed more potent activity than the 30%HCW group (Figure 3B). The 30%HCW and 50%HCW groups displayed significant activity for increasing the mammary index of female rats and their breast index levels (Figure 3C). Bromocriptine could significantly decrease the level of PRL, PRLR, and P; however, the 30%HCW and 50%HCW groups could overcome the effect of bromocriptine (Figures 3D–F), which indicated that both eluates could significantly increase the hormone level in female rats and promote lactation. In addition, the serum PRL levels of 30%HCW and 50%HCW groups were found to be similar in female rats (Figure 3D), but the PRLR level of the 50%HCW group was higher than that of the 30%HCW group (Figure 3E). The progesterone level of the 30%HCW group was higher than that of other treatment groups.

Compared to the control group, the model group showed severe atrophy of mammary tissues, and a large amount of hyperplastic connective and adipose tissue replaced the normal mammary tissue. After treatment with 30%HCW and 50%HCW, the area of the mammary lobules was increased. The lumen of the intrafollicular alveoli was significantly enlarged (Figure 3G). The level of prolactin receptors in the model group was significantly decreased compared with the control group (Figure 3H). Still, after treatment with 30%HCW and 50%HCW, the level of prolactin receptors was significantly increased. The above results indicated that 30%HCW and 50%HCW display significant lactation-promoting activity.

### 3.4 Identification of the main components of 30%HCW and 50%HCW by UPLC-Q/TOF-MS

The above result indicated that 30%HCW and 50%HCW display significant lactation-promoting activity by improving the hormone level in female rats; however, the specific functional ingredients are still unknown and need further investigation. In this study, the main ingredients in 30%HCW and 50%HCW were detected and identified by a UPLC-Q/TOF-MS method, which was a classic technology to identify the metabolites from medicinal plants or herbs. The main compounds, which display apparent peaks in the total ion chromatograms (TICs) of 30%HCW and 50%HCW, were primarily detected and grasped by their precise mass-to-charge (*m/z*) ratio. Then, the MS/MS spectra of the detected ingredients were obtained by the targeted MS/MS method. Finally, the structures of those main ingredients were identified by their MS/MS spectra and well-known fragmentation pathways of flavonoids and chlorogenic acid-type compounds (Liu et al., 2020) taking metabolites **11**, **12**, **18**, and **19** as an example to demonstrate how to identify the structures of main compounds.

**FIGURE 4:**
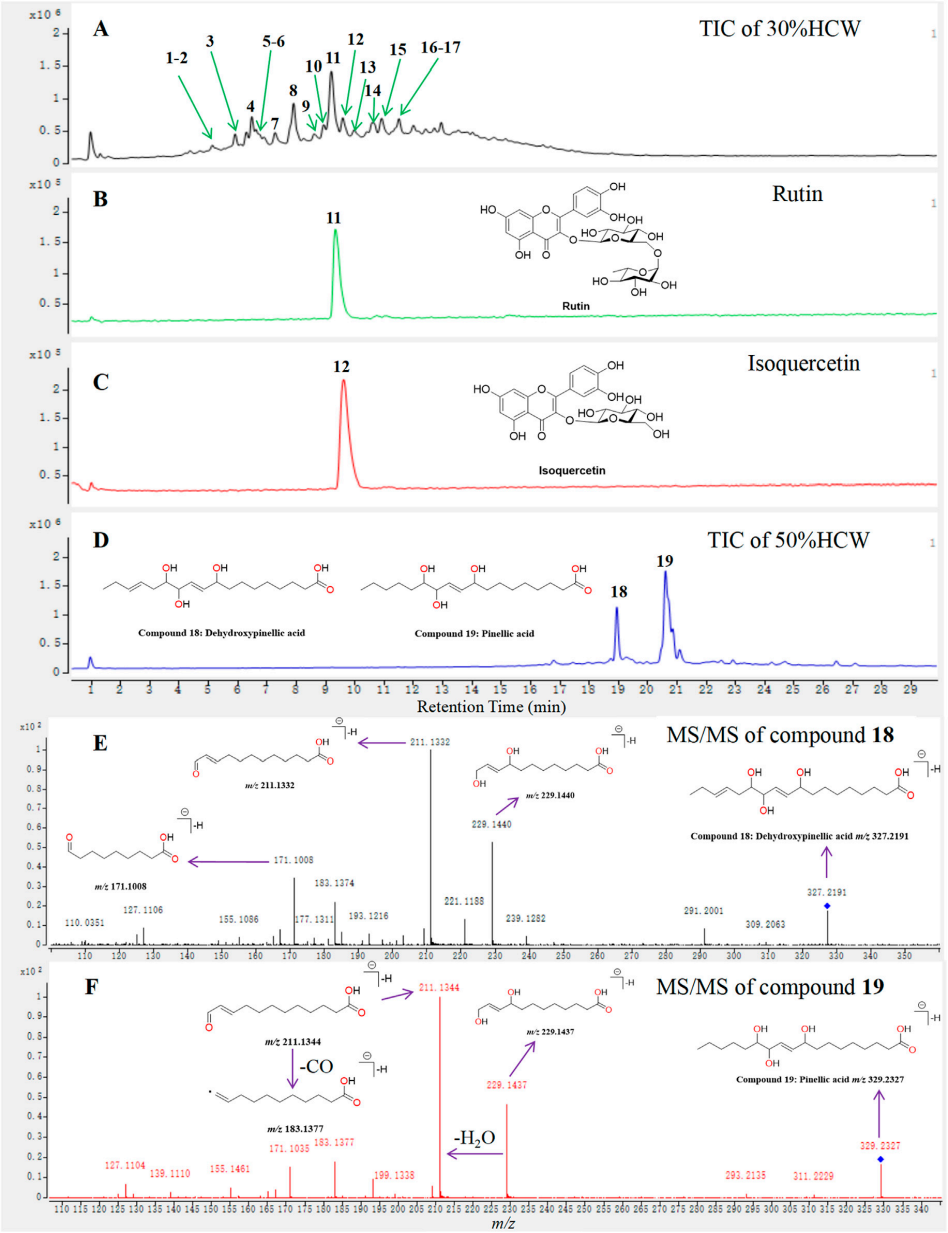
Identification of the main compounds of 30%HCW and 50%HCW by UPLC-Q-TOF-MS. **(A)** Total ion chromatogram (TIC) of 30%HCW; **(B)** TIC of rutin; **(C)** TIC of isoquercetin; **(D)** TIC of 50%HCW and the structures of compounds **18** and **19**; **(E)** MS/MS of compound **18** and corresponding fragmentation ions; and **(F)** MS/MS of compound **19** and corresponding fragmentation ions.

Compounds **11** and **12** were present in the TIC of 30%HCW (Figure 4A), and their deprotonated *m/z* values were 609.1459 and 463.0852, respectively. According to the MS/MS of both metabolites, the loss of two glucose and one glucose moiety from mother ions and forming the skeleton ions at *m/z* 300.0275 and 300.0290, respectively, could be observed (Supplementary Figure S1). These characteristic data indicate that compounds **11** and **12** were tentatively regarded as rutin and isoquercetin. Finally, compounds **11** and **12** were unambiguously identified as rutin and isoquercetin by comparing the retention time, MS, and MS/MS with the standards (Figures 4B, C). Compounds **18** and **19** were present in the TIC of 50%HCW (Figure 4D), and their deprotonated *m/z* values were 327.2191 and 329.2327, respectively. The characteristic fragment ions at *m/z* 229, 221, and 211 in the MS/MS of compounds **18** and **19** were used to screen the mass spectral database named MassBank (https://massbank.eu/MassBank/Search). The search results indicated that compounds **18** and **19** were dehydroxypinellic acid and pinellic acid, respectively. The structures of both compounds were further elucidated and identified by the fragment ions of MS/MS spectra (Figures 4E, F). Compounds **18** and **19** were reported for the first time from *H. citrina*. The other ingredients have also been identified by similar methods, and their corresponding information is shown in Table 1; Supplementary Figure S1.

**TABLE 1:** Main ingredients in 30%HCW and 50%HCW identified by the UPLC-Q-TOF-MS method.

Peak no.	*t*_R_ min	[M-H]^−^ *m/z*	Molecular formula	Relative content	MS/MS fragment ions (*m/z*)	Identification
1*	5.08	353.0878	C_16_H_18_O_9_	6.9%^a^	191.0526, 173.0381, and 135.0415	Chlorogenic acid
2	5.18	367.1032	C_17_H_20_O_9_	4.5%^a^	313.7033, 193.0490, and 135.0360	Methyl cryptochlorogenic acid
3*	5.92	337.0931	C_16_H_18_O_8_	11.2%^a^	191.0526, 164.0349, and 119.0514	3-*O*-p-coumaroylquinic acid
4*	6.54	337.0931	C_16_H_18_O_8_	17.9%^a^	173.0458, 163.0391, 155.0277, and 111.0452	4-*O*-p-coumaroylquinic acid
5^#^	6.91	461.1662	C_21_H_20_O_9_	4.7%^a^	269.1065 and 161.0456	Dihydroxykaempferol-3′-*O*-glucoside
6	7.31	367.1032	C_17_H_20_O_9_	5.6%^a^	193.0479, 173.0477, and 134.0365	Methylchlorogenic acid
7*	7.79	337.0931	0	13.8%^a^	191.0565 and 173.0408	5-*O*-*p*-coumaroylquinic acid
8	8.00	755.2040	C_33_H_40_O_20_	62.3%^a^	300.0279, 271.0254, and 178.9981	Quercetin-3-*O*-rutinose -(1→2)-*O*-rhamnoside
9	8.91	739.2085	C_33_H_40_O_19_	7.9%^a^	284.0327, 255.0286, and 178.9951	Kaempferol-3-*O*-rutinose- (1→2)-*O*-α-L-rhamnopyranose monohydrate
10	9.01	609.1462	C_26_H_28_O_17_	8.9%^a^	343.0367, 300.0277, 271.0242, 255.0305, 178.9927, and 151.0017	Isorutin
11*	9.35	609.1462	C_27_H_30_O_16_	100%^a^	301.0331, 300.0275, 271.0252, 255.0301, 178.9953, and 151.0033	Rutin
12*	9.71	463.0887	C_21_H_20_O_12_	20.5%^a^	300.0290, 271.0244, 255.0291, 178.9976, and 151.0026	Isoquercetin
13	10.05	579.1352	C_26_H_28_O_15_	8.9%^a^	300.0272, 271.0246, and 151.0020	Quercetin-3-*O*-rhamnose-(1→2)-arabinose
14	10.45	433.0783	C_20_H_18_O_11_	17.5%^a^	300.0231 and 271.0234	Quercetin-3-*O*-α-L-arabinoside
15	10.46	447.0931	C_21_H_20_O_11_	19.7%^a^	284.0324, 255.0291, and 183.0383	Astragalin
16	10.65	593.1510	C_27_H_30_O_15_	10.7%^a^	285.0387 and 151.0022	Nicotiflorin
17	10.99	623.1617	C_28_H_32_O_16_	1.9%^a^	315.0509, 300.0269, 271.0246, and 151.0009	Isorhamnetin-3-*O*-rutinoside
18^#^	24.81	327.2815	C_18_H_32_O_5_	29.0%^b^	229.1439, 211.1333, 171.1017, 155.1438, and 127.1125	Dehydroxypinellic acid
19^#^	26.36	329.2340	C_18_H_34_O_5_	100%^b^	311.2219, 229.1438, 211.1336, 171.1017, 155.1448, and 139.1123	Pinellic acid

*represents compounds that were unambiguously identified by comparing the retention time, MS, and MS/MS with the standards.

^#^represents those compounds that were reported for the first time from *H. citrina.*

^a^ represents the relative content in 30%HCW.

^b^ represents the relative content in 50%HCW. Rutin (**11**) and pinellic acid (**19**) are the highest content compounds in 30%HCW and 50%HCW, respectively. Therefore, the relative content of both compounds is identified as 100% in 30%HCW and 50%HCW, respectively.

### 3.5 Rutin (11) and isoquercetin (12) display significant lactation-promoting activity

**FIGURE 5:**
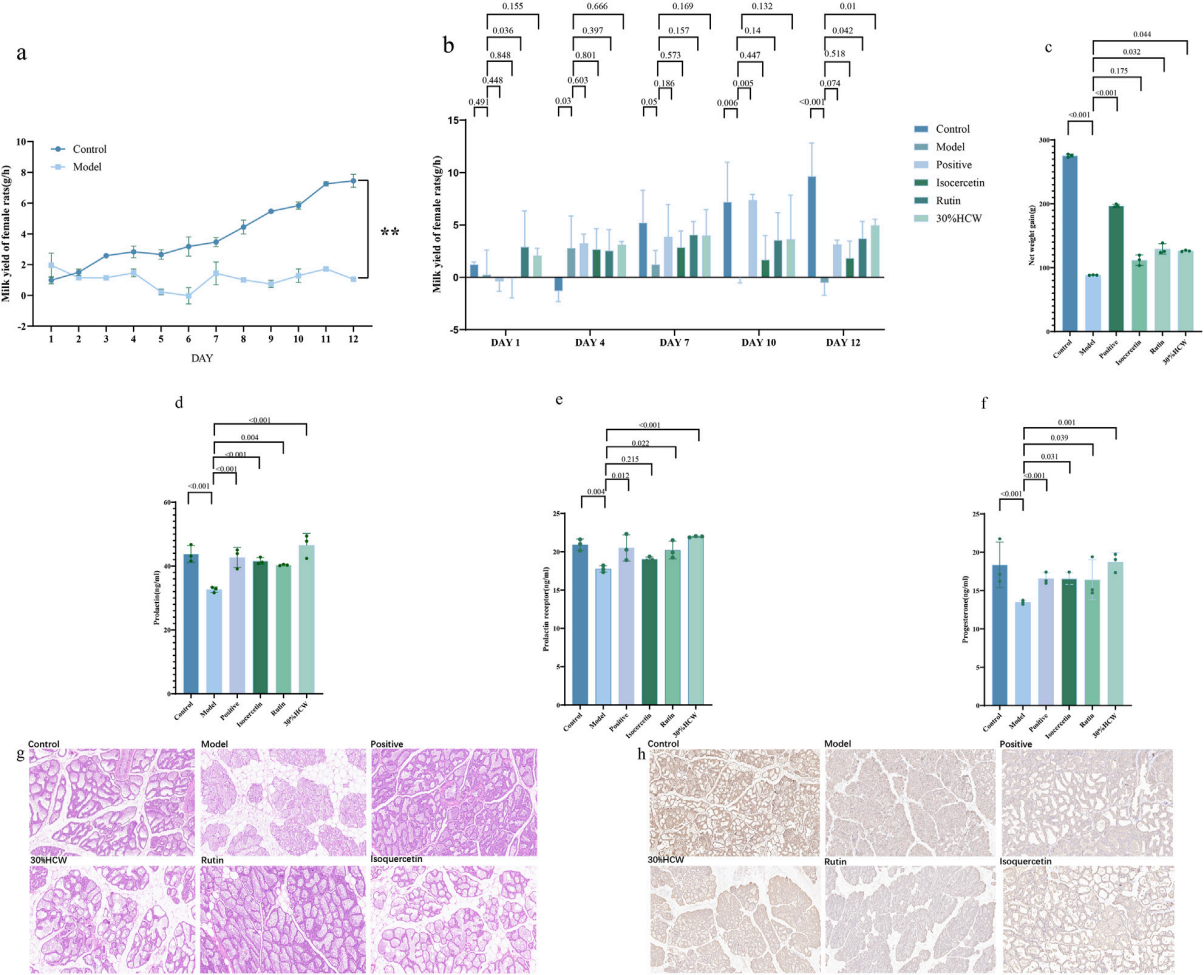
Lactation-promoting activity of rutin and isoquercetin. **(A)** Hourly lactation in the model and control groups; **(B)** hourly lactation in different groups of female rats on days 1, 4, 7, 10, and 12; **(C)** net weight gain of each groups; levels of PRL **(D)**, PRLR **(E)**, and P **(F)** in female rats; **(G)** HE staining of mammary tissue of different groups, 20 ×; and **(H)** immunohistochemical staining of PRLR in the mammary tissue of different groups.

After treatment with 4 mg/kg/day of bromocriptine for 12 days, the hourly milk production of female rats in the model group was significantly decreased compared with the control group (Figure 5A; *p* <0.01), which indicated that the *postpartum* lactation model was built successfully. The hourly milk production of female rats in the rutin (**11**) and 30%HCW groups significantly increased compared with the model group (Figure 5B; *p* < 0.05), which indicated that the rutin (**11**) and 30%HCW groups displayed significant lactation-promoting activity. As shown in Figure 5C, the positive drug, rutin, and 30%HCW groups could significantly increase the net weight gain compared with the model group (*p* < 0.001, 0.032, and 0.044, respectively), which also demonstrates that rutin and 30%HCW have significant lactation-promoting activity. In addition, isoquercetin (**12**) could increase the hourly milk production of female rats and the net weight gain compared with the model group (Figures 5B, C).

Bromocriptine significantly decreased the level of PRL, PRLR, and P (*p* <0.001, 0.004, <0.001, respectively; Figures 5D–F). Rutin (**11**) and isoquercetin (**12**) could overcome the effect of bromocriptine, which indicated that both compounds could significantly increase the hormone level in female rats. In addition, the serum PRL, PRLR, and P levels in rutin and isoquercetin groups were found to be similar in female rats, but those hormone levels in the 35%HCW group were higher than those in both compound and control groups, which indicated that rutin (**11**) and isoquercetin (**12**) were the main compounds responsible for the lactation-promoting activity.

The histopathological analysis of the mammary glands of female rats was conducted through HE staining. Under the microscope, the mammary gland of female rats in the control group displayed a neat and complete arrangement of mammary alveoli. In contrast, the mammary tissue of the model group was severely atrophied, with a significant amount of hyperplastic connective and fatty tissue replacing the normal mammary tissue (Figure 5G). Compared to the model group, the 30%HCW, isoquercetin, and rutin groups showed a larger area of restored lobules. The 30%HCW group had the most effective intervention on mammary tissue morphology, with mammary lobules tending toward normal. Immunohistochemical staining showed that the PRLR in the mammary gland was mainly concentrated in the alveoli and ductal cells (Figure 5H). Compared to the model group, the 30%HCW group showed the darkest yellow positive discoloration, followed by the isoquercetin and rutin groups. This suggested that the 30%HCW group had the highest amount of prolactin receptor protein distribution. The above experiment results indicated that 30%HCW, isoquercetin, and rutin could improve the level of PRLR in the mammary gland and display lactation-promoting activity.

## 4 Discussion

In ancient books, *H. citrina* was recorded to have the effects of “reducing swelling and stopping bleeding, sedation, diuretic, and promoting lactation.” *H. citrina* was also used as a traditional function food stewed with fish or chicken to promote milk secretion for *postpartum* women in China. In this study, it was found that the aqueous extract of *H. citrina* displayed a significant lactation-promoting effect; however, the alcoholic extract did not show apparent activity. These experiment results provide a scientific basis for using water as a solvent to cook *H. citrina* in folk.

*H. citrina* was used as a functional food for a long time in China; however, the recommended dose of edible *H. citrina* to promote milk secretion is still unknown. In this study, 1,000 and 2,000 mg/kg aqueous extract of *H. citrina* showed significant lactation-promoting activity. According to the mouse- (20 g) and human (70 Kg)-specific surface area conversion (Grosenbach et al., 2018) and the extraction rates of dried *H. citrina* (approximately 30%) (Liu et al., 2022), the theoretical recommended dose of edible *H. citrina* was 25–50 g/d for *postpartum* women to promote milk secretion.

The lactation-promoting activity of *H. citrina* extracts and dried powder has been evaluated in previous studies, and the results showed that both of them displayed significant effects (Zhong et al., 2021; Guo et al., 2023). However, the specific active ingredients for the corresponding activity are still unknown. In this study, 30%HCW and 50%HCW showed significant lactation-promoting activity, and their main compounds were identified by UPLC-Q-TOF-MS combined with the previous well-built MS database of *H. citrina* (Liu et al., 2020). Rutin (**11**) and isoquercetin (**12**) displayed significant lactation-promoting activity and were regarded as the main active ingredients of 30%HCW. This is the first study to demonstrate that rutin and isoquercetin are the lactation-promoting active ingredients. Dehydroxypinellic acid (**18**) and pinellic acid (**19**) were the main compounds of 50%HCW; however, it is difficult to obtain enough monomer components for the lactation-promoting experiments. Therefore, compounds **18** and **19** were regarded as the potential lactation-promoting compounds and need further study.

**FIGURE 6:**
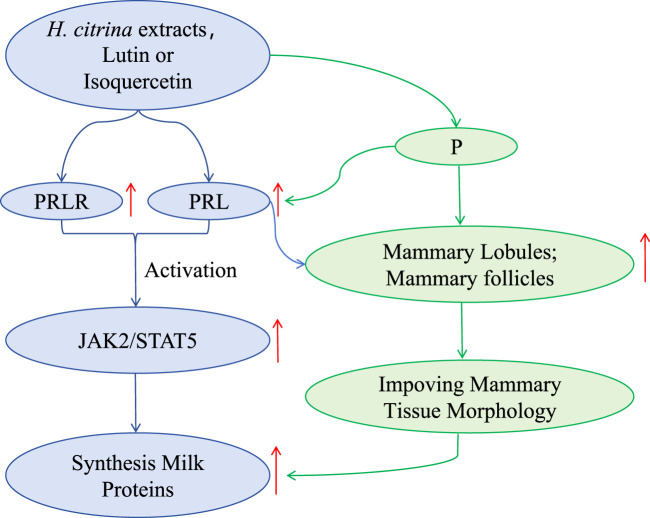
Possible lactation-promoting mechanism of *H. citrina* extracts or compounds.

In this study, the *H. citrina* aqueous extract, 30%HCW, 50%HCW, rutin, and isoquercetin could significantly improve the level of PRL, PRLR, and P for *postpartum* female rats. The increased PRL and PRLR combination could activate the kinase 2 (JAK2) and transcription 5 (STAT5) signaling pathways (Figure 6). The synthesis of breast milk proteins, such as β-casein and α-lactalbumin, will be enhanced after activation of the JAK2/STAT5 signaling pathway (Buntin and Buntin, 2014; Zhang et al., 2021; Guo et al., 2023). Additionally, it is generally accepted that PRL promotes the development of lobular follicles in the mammary gland and the proliferation of lobular epithelial cells during pregnancy (Hannan et al., 2023). Progesterone (P) is a major hormone in the maintenance of pregnancy, and its central role is to promote the development of mammary lobules and follicles and to exert its mammary gland-promoting effects (Berg et al., 2002; Jin et al., 2024). Therefore, the increased PRL and P levels could ameliorate the mammary lobules and follicles and improve the mammary tissue morphology, increasing the synthesis of breast milk (Figure 6). The potential mechanism is that the active ingredients present in *H. citrina* increase the levels of PRL, PRLR, and P and then exert the lactation-promoting activity by the above signaling pathway (Figure 6).

## 5 Conclusion

*H. citrina* was a traditional function food used for promoting lactation in *postpartum* mothers for a long time in China; however, the active ingredients and corresponding mechanisms are still unknown. In this study, the lactogenic effect of alcoholic and different doses of aqueous extracts of *H. citrina* was primarily evaluated, and the aqueous extract (1,000 and 2,000 mg/kg) displayed significant lactation-promoting effects. Then, three eluates of the aqueous extract (0%, 30%, and 50%HCW) further assessed their lactogenic effect; the result indicated that 30% and 50%HCW showed significant lactation-promoting activity. A total of 19 ingredients, including the main compounds named rutin, isoquercetin, dehydroxypinellic acid, and pinellic acid, were identified by the UPLC-Q-TOF-MS method from 30% and 50%HCW. Finally, the lactogenic effect of rutin and isoquercetin was evaluated, and both of them were identified as the lactation-promoting active ingredients of *H. citrina*. The corresponding mechanisms relative to the lactation-promoting activity for *H. citrina* extracts and monomer components are to stimulate the expression and release of PRL and P, as well as to induce the expression of PRLR in the mammary tissue. In addition, 30%HCW, 50%HCW, rutin, and isoquercetin could significantly improve mammary tissue morphology. This study first clarified the lactation-promoting active ingredients of *H. citrina* and provided lead compounds to the new lactation-promoting drug.

## Data Availability

The original contributions presented in the study are included in the article/Supplementary Material, further inquiries can be directed to the corresponding authors.
