# Supplementary material for: Lactation-promoting ingredients of Hemerocallis citrina Borani and the corresponding mechanisms
Source: Front Pharmacol. 2024 Sep 23;15:1431856. doi: 10.3389/fphar.2024.1431856 (PMC11456526; doi:10.3389/fphar.2024.1431856)
Supplement: Supplementary file 1 [file DataSheet1.docx]

Supplementary Material

# Supplementary Data


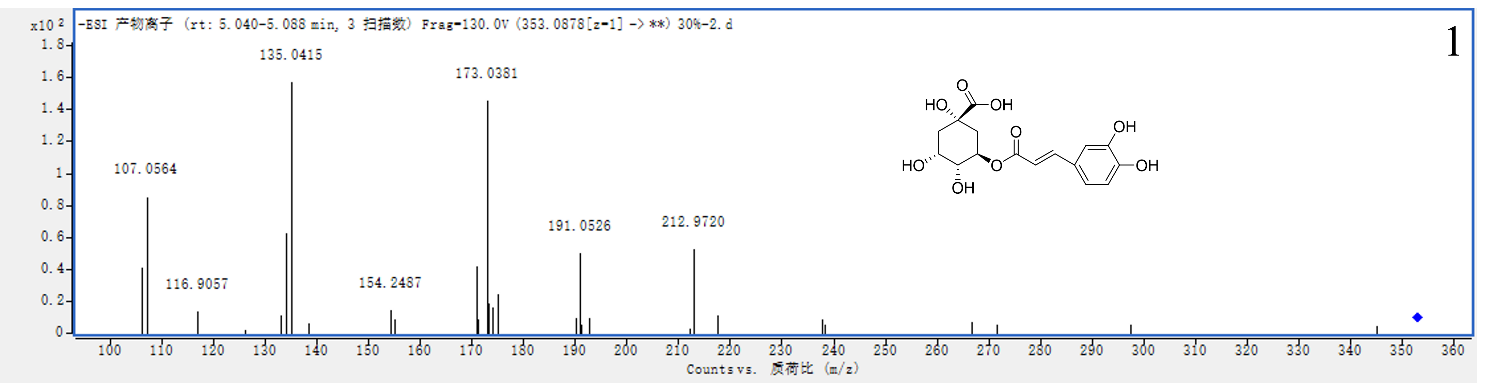


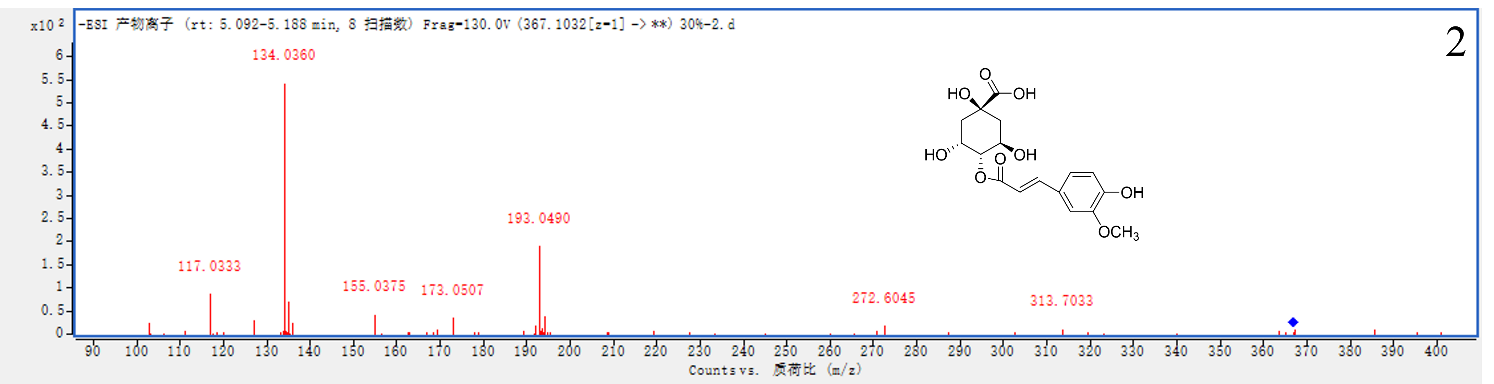


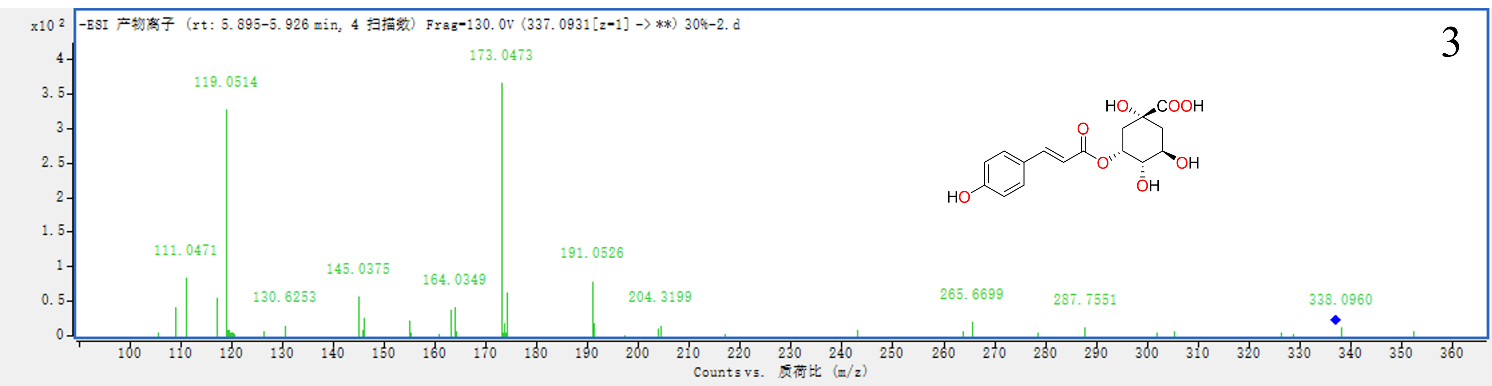


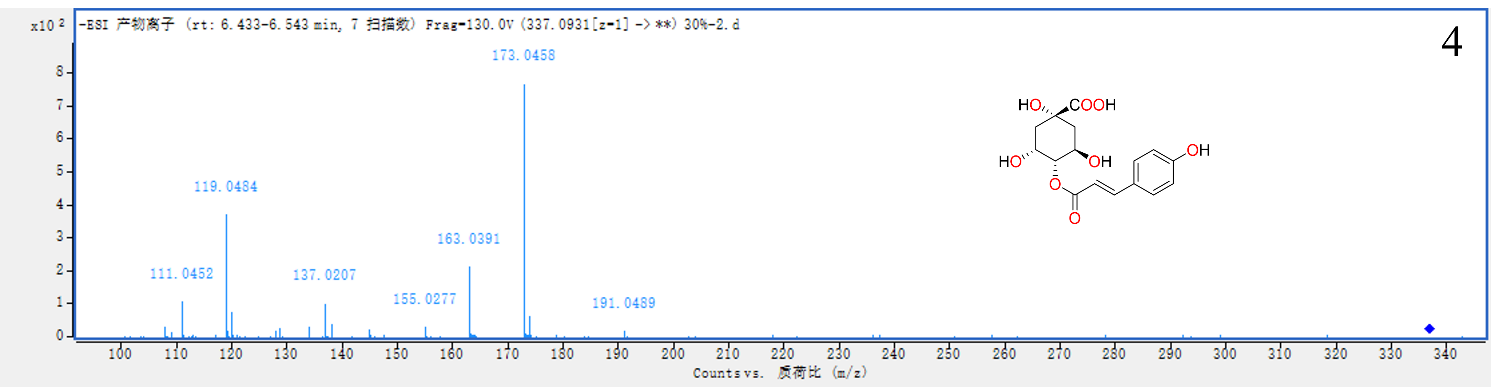


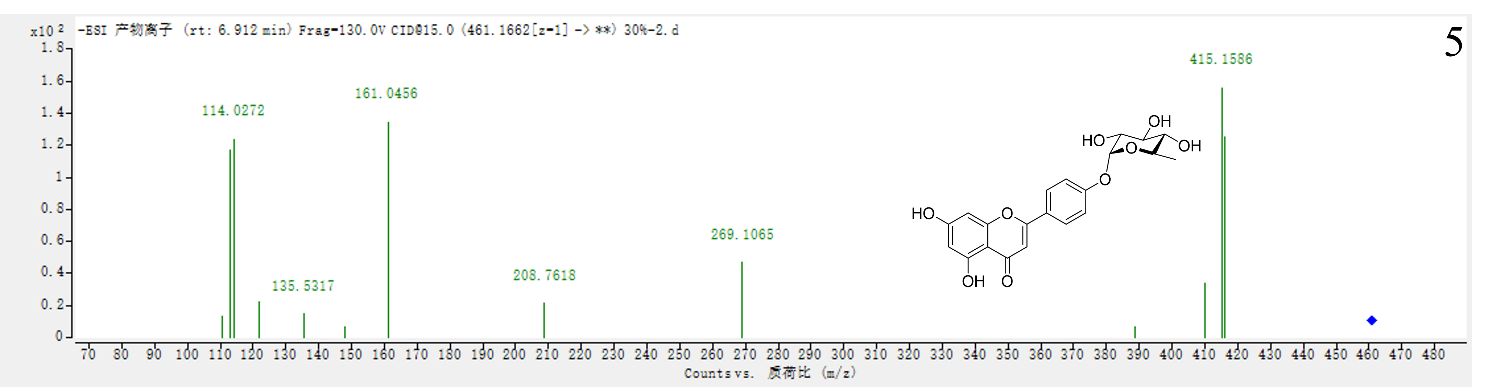


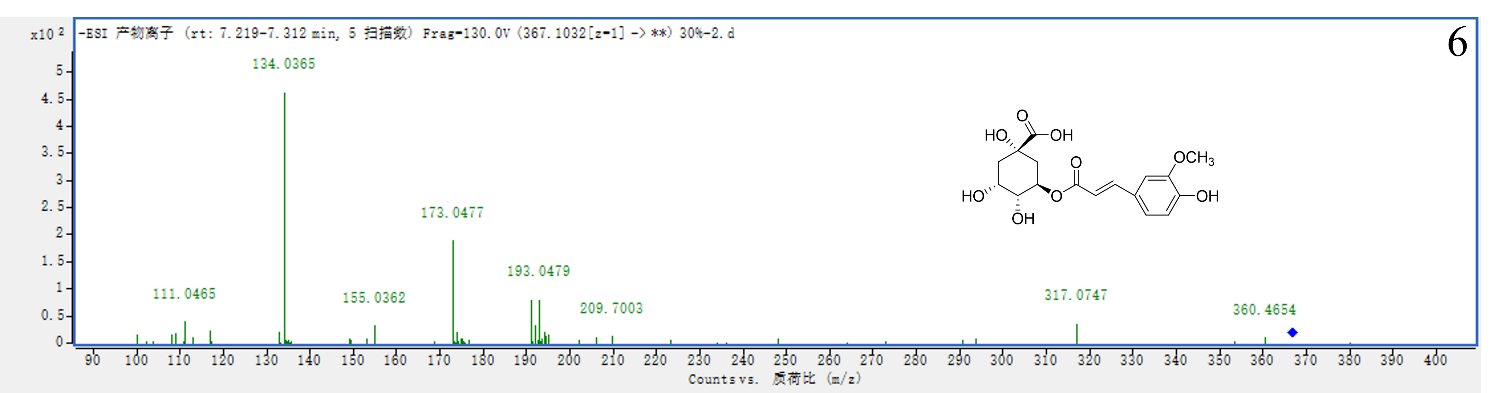


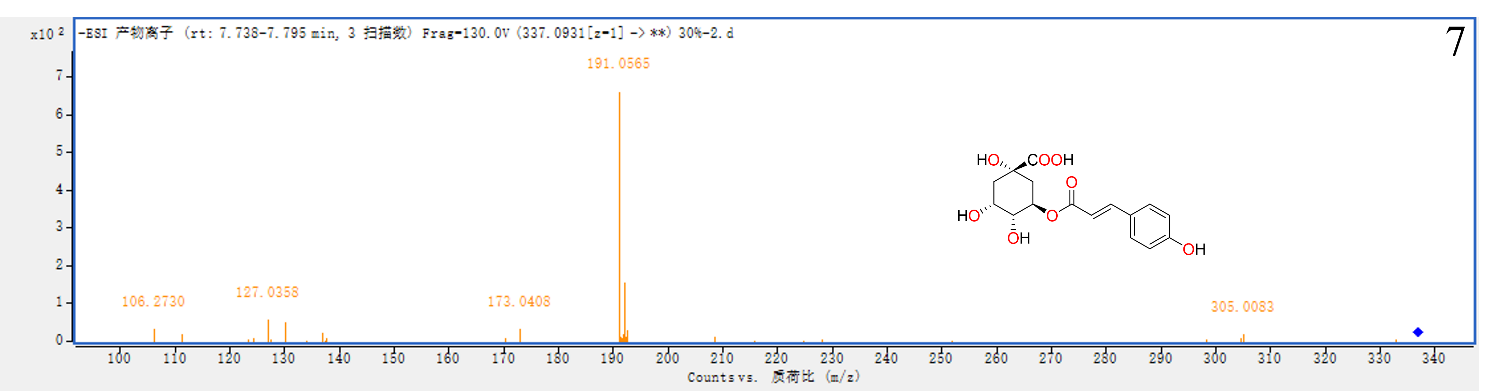


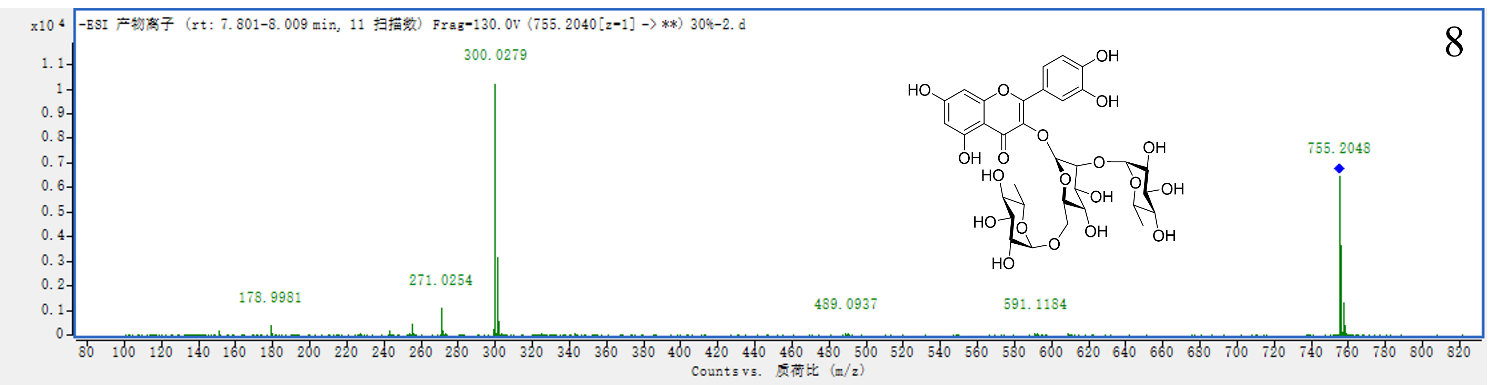


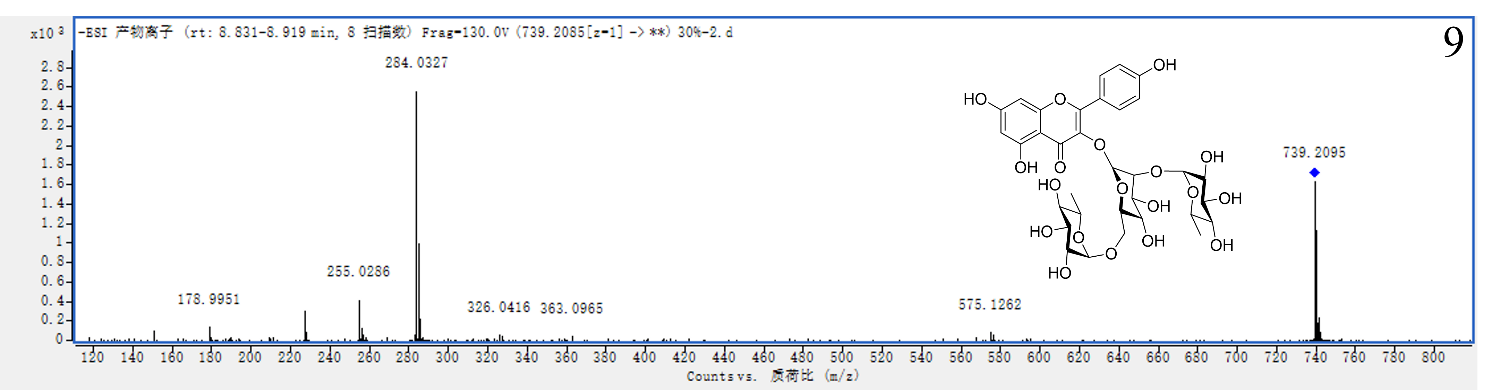


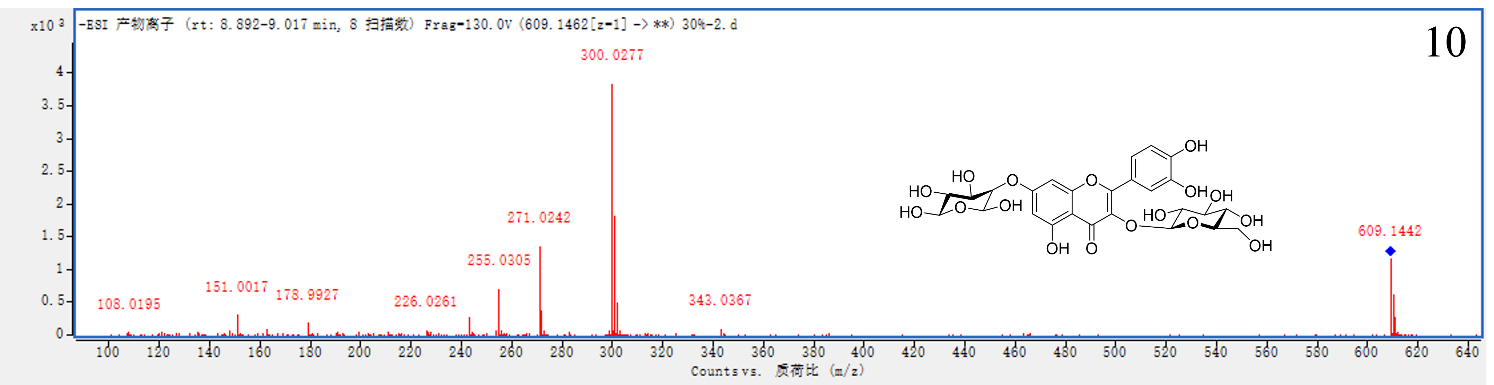


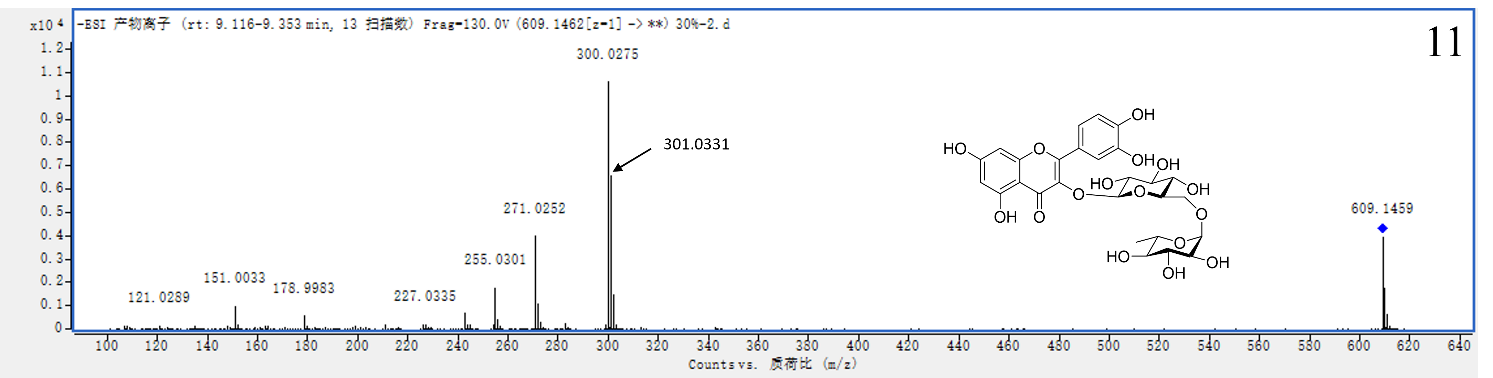


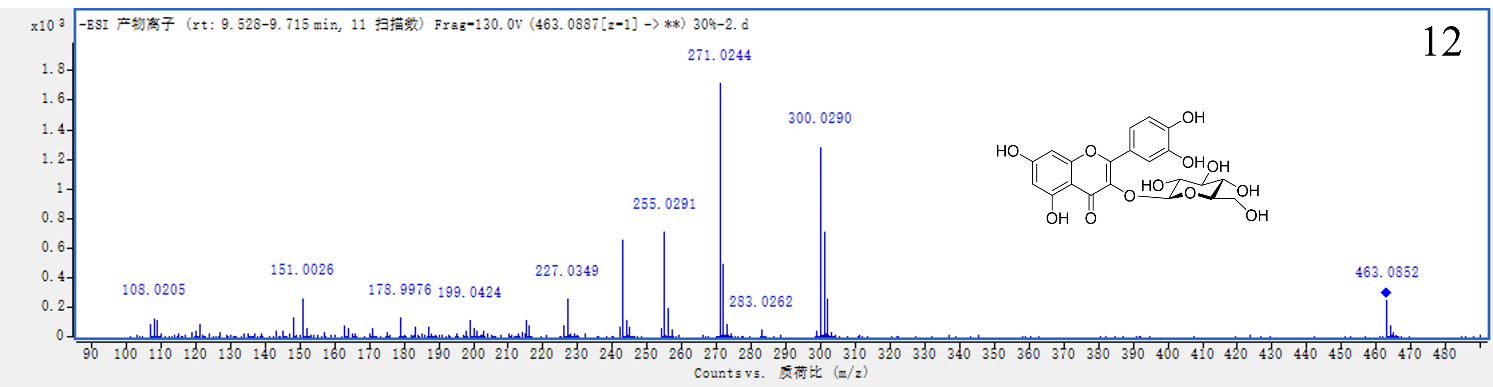


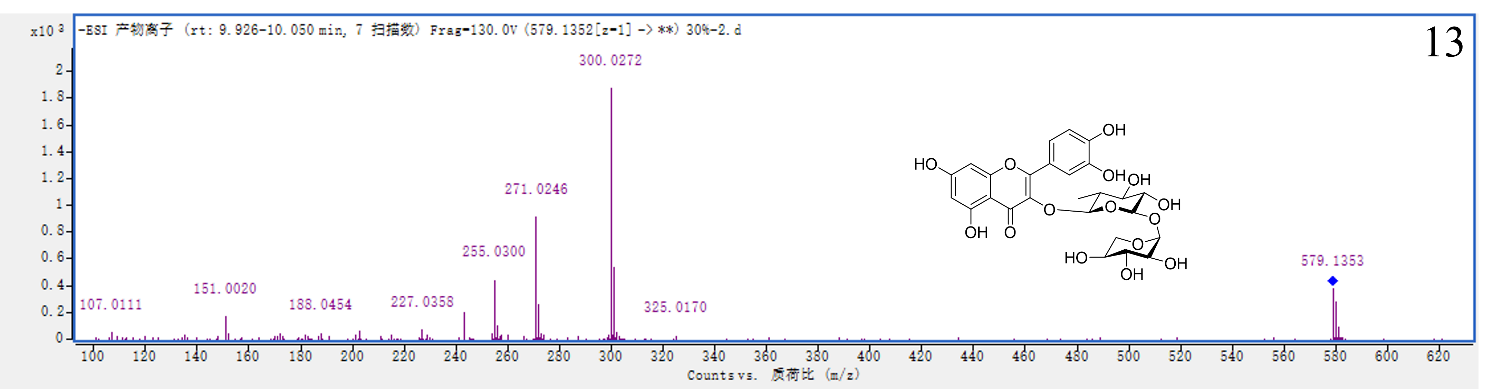


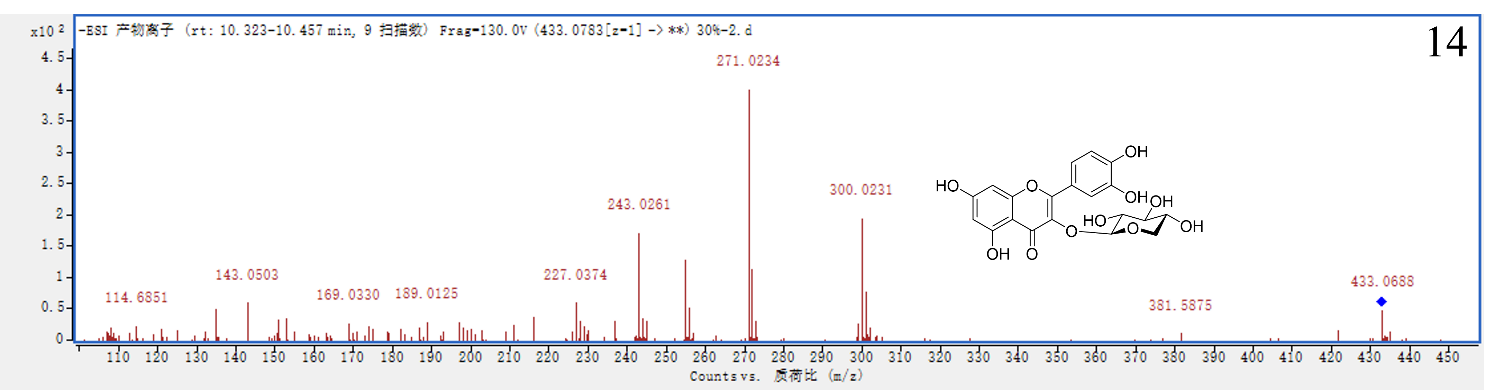


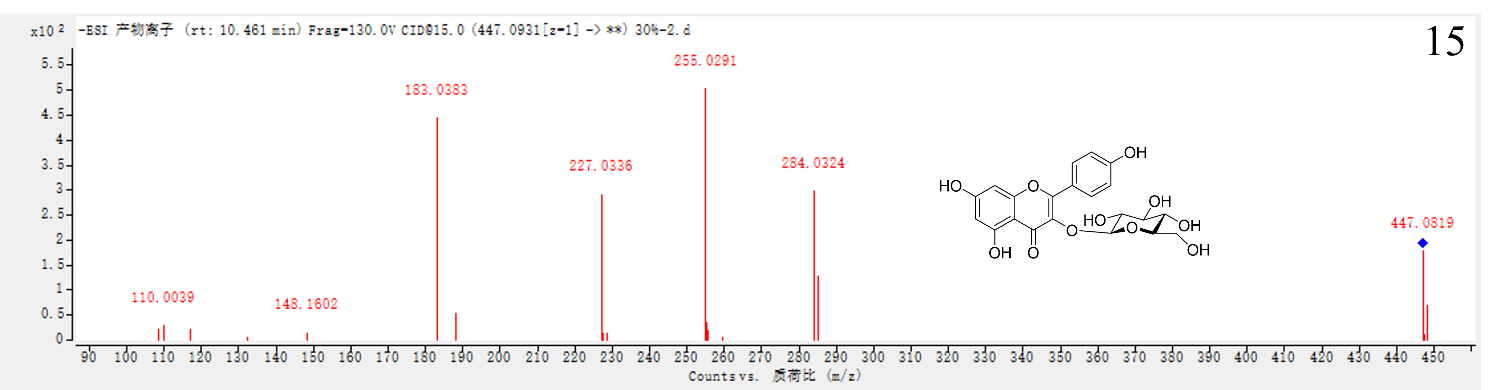


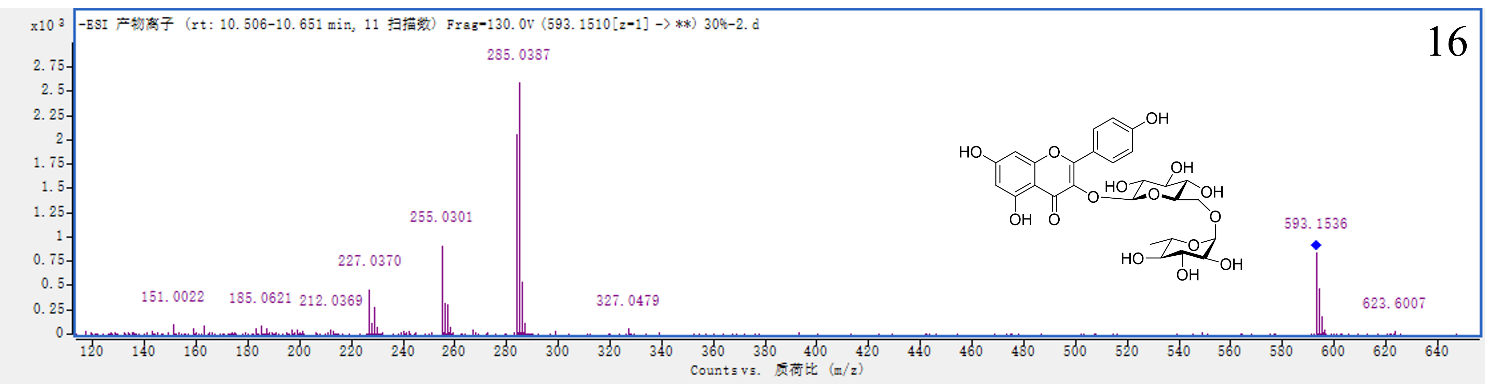


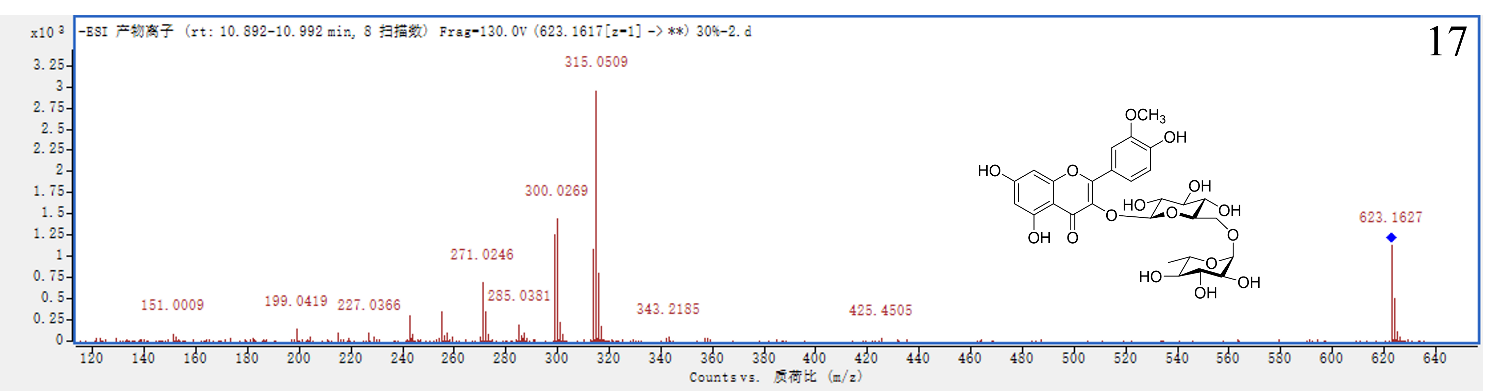


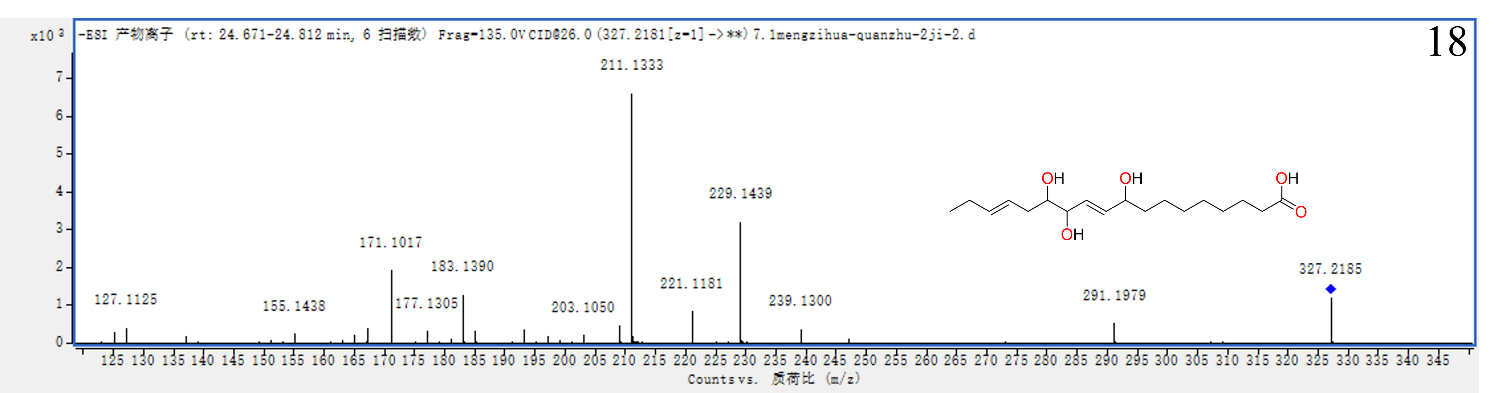


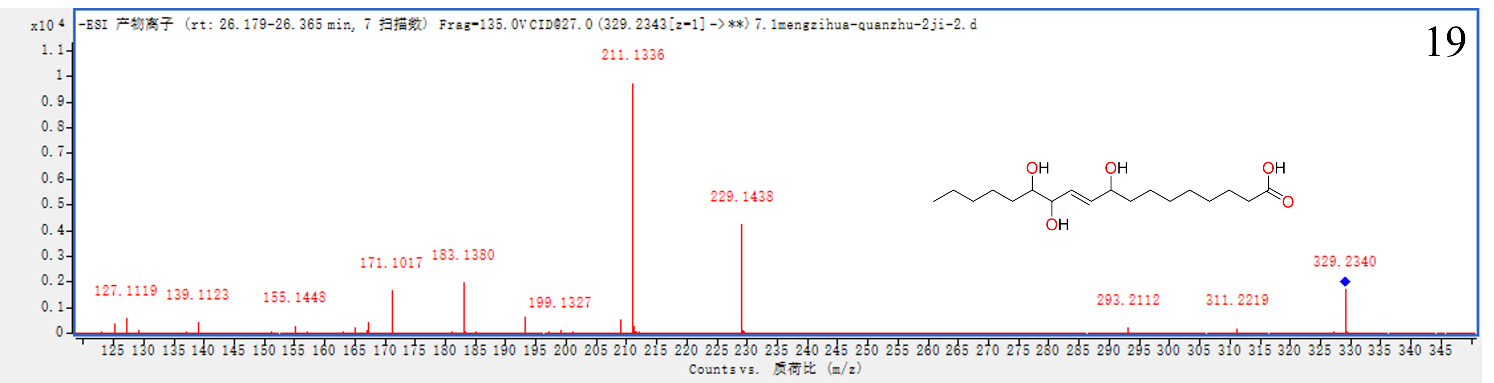


MS/MS of identified metabolites of 19 different fractions of Hemerocallis citrina Borani
